# Conflict or consensus? Re-examining crime and disaster

**DOI:** 10.4102/jamba.v11i1.744

**Published:** 2019-08-19

**Authors:** Bethany L. van Brown

**Affiliations:** 1 Department of Sociology and Criminolgoy, Cabrini University, Pennsylvania, United States

The debate about whether or not crime – specifically looting – happens after disaster is an enduring debate in the field of disaster studies. Veteran disaster scholars argue that widespread looting only happens during a civil disturbance (riot), because it is a social conflict situation. Conversely, the same scholars say that widespread looting is rare and isolated during disaster, because it is a consensus situation. That is, during disaster, people act pro-socially and help each other (rescue, supplies, shelter), not antisocially and against each other (crime). However, I argue we need to revisit this claim, because our social world is very different from what it was when this research was conducted. Social problems like inequality are more pronounced, and there is widespread social unrest. I contend that firstly, there is evidence of widespread looting during certain disaster events as well as civil disturbances, and secondly, the looting that happens during both civil disturbances and disasters is similar because those who are engaging in such behaviour share similar motives – the belief that there is no other way to voice their grievances than to riot.

The Halifax ship explosion in 1917 (Prince 1920) prompted scientific inquiry into human behaviour after disaster and was one of the starting points for the sociology of disaster subfield. Shortly thereafter, Sorokin (1942) examined the individual and society during what he called calamity. Both of these seminal works claimed that because disasters incite social change, the home discipline that made the most sense was sociology. Over the next couple of decades a large body of research emerged on human behaviour during disaster that embraced a sociological perspective. However, there are gaps in this body of knowledge, and as Frailing and Harper (2017) tell us, criminology has much to offer with regard to understanding antisocial behaviour during disaster.

One challenge in furthering our understanding of human behaviour during disaster is the definition of the term disaster. Fritz (1961) authored one of the earliest conceptualisations of the term, yet consensus on the definition of disaster is still tenuous and depends on who is defining the term. A disaster scholar likely has a different definition than an emergency management practitioner, who has a different definition than a policymaker and so on. Scholars from a variety of disciplines have all made invaluable contributions to how we conceptualise the term disaster, and for the last several decades the trend has been to focus on the social nature of disaster and the interaction between society and the environment, rather than physical aspects (Kreps 1998). As Quarantelli (2005:339) contends, ‘disaster is rooted in the social structure or social system’.

Disaster sociologists have compiled extensive evidence of pro-social behaviour during disaster, showing that the norms that emerge in the wake of disaster support a notion of helping and collective support for survival and recovery. Overwhelming evidence from these early disaster studies demonstrates that pro-social behaviour is more common than antisocial behaviour. However, there is increasing evidence that antisocial behaviour does happen during disaster, despite the inherent methodological challenges in measuring post-disaster behaviour. Following Hurricane Hugo, researchers from the Disaster Research Center conducted field studies to measure the extent of looting, including a quantitative survey of all businesses in major shopping centres (Quarantelli 1994). Findings revealed that massive looting occurred in the wake of Hugo, and while Quarantelli does acknowledge that widespread looting could be because of pre-existing social inequality, this idea has not been fully explored. Frailing and Harper (2016) examined looting that followed Hurricane Katrina by examining burglary rates in the month before and the month after the flood. They too found patterns of looting that contradict earlier findings that looting is rare. Here it is important to note that the official responses to Katrina as well as Hugo may have been criminogenic, and the looting that occurred after Katrina may have been in part the result of slow responses. For example, the island of St. Croix was absolutely crippled by Hurricane Hugo, and people did not know where to turn. This, combined with pre-existing inequalities, suggests potential motivations for widespread looting (Quarantelli 2008). The heavy pockets of concentrated poverty in the city only compounded disaster survivors’ angst, fuelled the disenfranchisement fire and may have just been the perfect storm, so to speak, for widespread looting to occur. Interestingly, there was not widespread looting following Hurricane Sandy, where the official response was more efficient.

Findings that show looting has happened during certain events have reignited the debate about whether or not crime – specifically looting – happens after disaster. This again points to an important conceptual clarification: the difference between a disaster and a civil disturbance, in this case a riot. Veteran disaster scholars argue that widespread looting only happens during a civil disturbance (riot), because it is a social conflict situation (Quarantelli & Dynes 1977). Conversely, the same scholars say that widespread looting is rare and isolated during disaster, because a disaster is a consensus situation. That is, during disaster, people act pro-socially and help each other (rescue, supplies, shelter), not antisocially and against each other (crime). In these findings, disaster survivors condemn looting because emergent norms elicit a therapeutic community where people are far more likely to help each other. This is different from the looting/property damage that happens during riots, where the behaviour is widespread and encouraged by others who are participating in order to send a message about property (e.g. rights).

I argue that we need to more carefully look at patterns of and explanations of looting in both riots and disasters because our social world is very different from what it was when the original disaster research was conducted. I am making the case that looting can happen during disaster and that the motivations for the looting that happens during disaster are similar to the motivations that cause a riot – like Quarantelli said, the reasons are rooted in the social structure and social systems. I am arguing that the looting that happens in both a disaster and a riot is sending a message about property, and that functions as a proxy for rights. Social problems like inequality are more pronounced, and there is widespread social unrest. We live in a political climate with extremely polarising rhetoric and where people feel emboldened to express various forms of discrimination against minority groups. Climate change is contributing to more frequent and severe disasters that are atypical, which is only exacerbating pre-existing environmental and social injustices. These injustices are erupting more often in the form of civil disturbances as a response to police brutality, and there is evidence of looting patterns during certain weather disasters that more closely resemble those found in civil disturbances.

The worsening social problems reflect increasing social vulnerability, an important variable in understanding the impact of disaster (Wisner et al. 2004). Sources of vulnerability intersect such that there are measurable patterns, which allow predictions to be made about what locations are most vulnerable to loss from disaster. Socio-economic status, household profile, income, education and other socio-economic and demographic variables create different levels of vulnerability for different groups of people. Cutter, Boruff and Shirley (2003) found the most common indicators of social vulnerability and created the Social Vulnerability Index (SoVI). In an effort to revise the SoVI, in 2013 Cutter included additional variables such as family structure, availability of transportation, ability status and access to health care. The SoVI is an invaluable contribution to understanding the social dimensions of disaster. Marginalised groups, then, are more vulnerable to disaster, and their vulnerability is rooted in underlying social processes. In addition to the SoVI, the Pressure and Release model (PAR) is a tool that is used for understanding how disasters happen when natural hazards affect vulnerable people (Wisner et al. 2004). The basis for the PAR model is that a disaster happens when two opposing forces intersect: social vulnerability and the natural hazard. The explanation of vulnerability has three sets of links that connect to the disaster process: root causes, dynamic pressure and unsafe conditions. Root causes reflect the exercise and distribution of power in society, and manifest as economically marginalised people. Dynamic pressures are economic, social and political processes that translate the effects of root causes into unsafe conditions, such as dangerous location and poor housing stock. The ‘release’ idea means that in order to relieve the pressure, vulnerability has to be reduced. The PAR model does not consider how cumulative disadvantage also cultivates anger in entire groups of people, which is one of the main ingredients in the commission of crime –particularly looting – during disaster.

Veteran disaster scholar Quarantelli (2008) established that mass looting occurred in St. Croix following Hurricane Hugo. Mass looting also occurred in the 1977 New York City blackout, which Quarantelli (2008:884) says is ‘a puzzling finding, for which no explanation has been offered by anyone to date’. There are several valid accounts of a wide range of criminal behaviour after Hurricane Katrina (e.g. Brown, Jenkins & Wachtendorf 2010; Harper & Frailing 2016). Quarantelli (2008:6) claims that these findings are ‘dubious’, although he does submit that the fact that these disasters are atypical may explain why crime occurred. This is precisely my point: Katrina was nothing if not an atypical disaster. And, if we are true to our social science definition of disaster, ‘it is the characteristic of the habitat being struck rather than the character of the force doing the striking that we study to get the measure of disaster’ (Freudenburg, Grambling & Laska 2009:6).

Disaster experts Quarantelli and Dynes (1977:8) say, ‘unlike the looting after disasters, looting in civil disturbances (riots) conveys an important message from the deprived sectors of the population’. What they mean is that looting during a civil disturbance is a way for disenfranchised populations who are upset to voice their grievances against an entire system of oppression. The looting, then, is a deliberate and symbolic attack. While these scholars acknowledge that looting may happen on rare occasions during a disaster, they argue that it is quite different from the looting that happens during a civil disturbance. I disagree with this claim and contend that firstly, there is evidence of widespread looting during certain disaster events as well as civil disturbances, and secondly, the looting that happens during both civil disturbances and disasters is similar because those who are engaging in such behaviour share similar motives – the belief that there is no other way to voice their grievances than to riot. As Martin Luther King Jr. said, ‘a riot is the language of the unheard’, (2013:1) which is similar to what Quarantelli and Dynes (1977) are saying. Civil disturbances, then, are intended to be an instrument for social change and are a way of voicing anger and disenfranchisement.

Civil disturbances have for a long time been situations of temporary and localised redefinitions of property rights (Quarantelli & Dynes 1968). Looting during civil disturbances involves protestors who loot property left vulnerable by owners fearing violence. The looting happens because the protestors feel that there is no other option for them to be heard, and property serves as a proxy for rights. Rights are not tangible, but property is. Property represents a shared understanding about who can choose to do what with the valued resources in a community. In other words, looting occurs owing to inequality. Looting is a mechanism to express anger towards the oppressor or oppressive system and serves as an attempt to redefine not only property rights but also basic human rights (Quarantelli & Dynes 1968).

The event – be it an instance of police brutality or a hurricane – is the tipping point that unleashes a long-simmering anger. For example, disasters that happen in places where there are heavy pockets of concentrated poverty, high unemployment rates, low median income and levels of education can also be situations of temporary and localised redefinitions of property rights because of the cumulatively disenfranchised populations and temporary breakdown in social organisation. It is no coincidence that these are the places where instances of police brutality are prevalent, too. This is not criminalising poverty, but instead is pointing to the root causes of why these populations are so desperate to be heard. The sudden disruption of a weather disaster also offers an opportunity to voice anger. This is different than saying people are engaging in opportunistic criminal behaviour, because they are not. The disruption of the disaster creates a perceived fracture in the social structure or system of power, which then creates the opportunity for disenfranchised people to express their grievances against the oppressor.

The literature tells us that in a disaster, there is general agreement among community members about community goals, especially about saving lives. Overall, a disaster is a consensus situation. In fact, in this body of work, there is found to be a therapeutic community that emerges, where people basically take care of one another until community functions are restored. While this may be true, these findings cannot necessarily be applied to all disasters and certainly cannot be applied to disasters of late. What’s more, if a disaster represents a consensus situation, for whom is a disaster a consensus? Certainly not everyone, and to apply this blanket assumption to all people in all communities runs the risk of overlooking some very important nuances about social inequality.

These places where there is evidence of looting after disaster – New Orleans, St. Croix, Haiti, New York City – also have in common many socio-demographic characteristics that are indicative of chronic inequality and social strain: high rates of concentrated poverty, high unemployment, high child poverty rates, a high number of female-headed households, low median household incomes and low literacy rates^1^.

Socio-demographic characteristics of Baltimore and Charlotte echo similar struggles, which support the hypothesis that a disaster may serve the same function as an incident of police brutality or a verdict in a trial – it is the tipping point that sparks marginalised groups to act out and express their anger, thereby making it a conflict situation as a result of long-time subjugation. These communities were under stress prior to the events, which then pushes marginalised populations living with cumulative structural racism and economic distress to express their anger.

The property damage in New Orleans, New York City, St. Croix, Haiti, Baltimore, and Charlotte resulted from decades of racism. Howard contends that there are lessons to be learnt from the riots, or what she calls urban uprisings, particularly that these more recent uprisings are not peculiar events, as they have been brewing for a long time (Schmidt 2015).

Civil disturbances, just like disasters, do not happen all of a sudden, although it is more comfortable for many people to think of them in that way. The violence comes after many failed non-violent attempts to make social change, yet that goes, unacknowledged too. Katrina was a ‘man-made’ disaster just as much the Ferguson or Baltimore civil disturbances were ‘man-made’, because inequality is man made. Really what Katrina did was lay bare all of the cumulative social and environmental injustices that existed long before the levees broke. Instead of addressing the root causes of inequality, the pot of grievances in marginalised communities is allowed to simmer until it boils over in the form of a civil disturbance that a verdict *or* weather event can trigger. Acknowledging and examining the similar patterns between these types of events will prohibit us from ignoring the complete pre-event context and the root causes of inequality. We must acknowledge the community characteristics where looting occurs in order to address root causes of inequality.

The news is saturated with stories of riots being constructed as ‘black rage’, which perpetuates long-lasting stereotypes of black people as criminals. These stories also fail to acknowledge the complete pre-existing social context that lead people to engage in this behaviour of last resort in the first place (Schmidt 2015). If we simply label rioters as criminals or ‘thugs’, we remove their political agency, which functions to maintain the status quo (Schmidt 2015). Collective denial about chronic inequality led to gross injustices well before the levees broke and police killed Freddie Gray. And it seems that with each uprising the subjugated people fighting for social change are getting fewer concessions. Marginalised groups will continue to use this tactic until meaningful, macro-level changes happen that relieve pressure and reduce vulnerability.

We still have much to learn about human behaviour during disaster, and the inherent challenges in measurement remain, but thankfully veteran disaster scholars have laid a strong foundation for us to continue this important work. This paper contributes to our understanding of the pre-disaster conditions that may or may not enable certain crimes by suggesting that there are similarities between the motives behind looting during a disaster as well as the motives of a riot. The field of disaster studies can benefit from explanatory work and can achieve empirical work that predicts when we may see certain types of crime in the wake of disaster.

## Footnotes

1 See, for example, U.S. Census Quick Facts: New Orleans https://www.census.gov/quickfacts/fact/table/neworleanscitylouisiana/PST045217
